# Purification of synchronized *Escherichia coli* transcription elongation complexes by reversible immobilization on magnetic beads

**DOI:** 10.1016/j.jbc.2022.101789

**Published:** 2022-03-03

**Authors:** Skyler L. Kelly, Courtney E. Szyjka, Eric J. Strobel

**Affiliations:** Department of Biological Sciences, The University at Buffalo, Buffalo, New York, USA

**Keywords:** transcription, RNA polymerase, DNA, RNA, transcription elongation complex, synchronized transcription, photocleavable biotin, solid-phase transcription, C3, capture sequence 3, etheno-dA, 1,N^6^-etheno-2′-deoxyadenosine, NTP, nucleoside triphosphate, P, pellet, ppGpp, guanosine-3′,5′-bisdiphosphate, RNAP, RNA polymerase, RT, readthrough, S, supernatant, SC1, structure cassette 1, T, terminated, TEC, transcription elongation complex, ZMP, 5-aminoimidazole-4-carboxamide-1-β-D-ribofuranosyl 5′-monophosphate

## Abstract

Synchronized transcription elongation complexes (TECs) are a fundamental tool for *in vitro* studies of transcription and RNA folding. Transcription elongation can be synchronized by omitting one or more nucleoside triphosphates from an *in vitro* transcription reaction so that RNA polymerase can only transcribe to the first occurrence of the omitted nucleotide(s) in the coding DNA strand. This approach was developed over four decades ago and has been applied extensively in biochemical investigations of RNA polymerase enzymes but has not been optimized for RNA-centric assays. In this work, we describe the development of a system for isolating synchronized TECs from an *in vitro* transcription reaction. Our approach uses a custom 5′ leader sequence, called capture sequence 3-structure cassette 1 (C3-SC1), to reversibly capture synchronized TECs on magnetic beads. We first show, using electrophoretic mobility shift and high-resolution *in vitro* transcription assays, that complexes isolated by this procedure, called ^C3-SC1^TECs, are >95% pure, >98% active, highly synchronous (94% of complexes chase in <15s upon addition of saturating nucleoside triphosphates), and compatible with solid-phase transcription; the yield of this purification is ∼8%. We then show that ^C3-SC1^TECs perturb, but do not interfere with, the function of ZTP (5-aminoimidazole-4-carboxamide riboside 5′-triphosphate)-sensing and ppGpp (guanosine-3′,5′-bisdiphosphate)-sensing transcriptional riboswitches. For both riboswitches, transcription using ^C3-SC1^TECs improved the efficiency of transcription termination in the absence of ligand but did not inhibit ligand-induced transcription antitermination. Given these properties, ^C3-SC1^TECs will likely be useful for developing biochemical and biophysical RNA assays that require high-performance, quantitative bacterial *in vitro* transcription.

In the past several years, bacterial transcription systems have been used to develop high-resolution biochemical and biophysical methods for studying RNA folding and function (1, 2, 3, 4, 5, 6, 7, 8, 9, 10, 11, 12, 13, 14). In many cases, biochemical tools that were developed for studying the process of bacterial transcription have been successfully repurposed for these applications. Among these tools, strategies for synchronizing transcription (15) are particularly important because they generate a virtually uniform population of transcription elongation complexes (TECs) that can be used in biochemical assays.

It is well-established that transcription complexes can be synchronized *in vitro* by ‘walking’ RNA polymerase (RNAP) to a synchronization site (15, 16, 17). In this approach, one or more nucleoside triphosphates (NTPs) are omitted from a transcription reaction so that RNAP transcribes until it reaches the first occurrence of the nucleotide that was omitted. Transcription then resumes when the NTP that was omitted is added to the transcription reaction. While transcription complexes are occasionally synchronized using natural DNA sequences, it is more common to initiate transcription using one of several established leader sequences that facilitate the formation of stable, synchronized TECs. This approach is applied routinely in high-resolution analyses of transcription elongation kinetics (15). For this application, the sequence of the 5′ leader and the purity of the synchronized TECs are typically inconsequential as long as the complexes are stable and resume transcription synchronously. However, when preparing synchronized TECs for assays that measure RNA folding or function, the sequence of the 5′ leader is crucial because it must be sequestered from interactions with the downstream RNA of interest. Furthermore, in some cases, the ability to purify synchronized TECs may be advantageous because it establishes a quantitative relationship between template DNA molecules and their RNA product. A standardized system for generating high-purity synchronized TECs for use in RNA biochemical assays could therefore aid the development of new technology for investigating RNA structure and function.

In this work, we describe a procedure for isolating synchronized *Escherichia coli* TECs from an *in vitro* transcription reaction using a 33 nt 5′ leader sequence, called capture sequence 3-structure cassette 1 (C3-SC1). Our approach builds upon established procedures for synchronizing bacterial transcription by integrating an oligonucleotide hybridization site and a fast-folding RNA hairpin with an A-less cassette that can be used to walk RNAP to a synchronization site. Together, these elements facilitate the purification of synchronized TECs using a straightforward reversible immobilization procedure and minimize potential interactions between the C3-SC1 transcript and downstream RNA nucleotides by sequestering 31 of 33 5′ leader nucleotides. We first show that TECs isolated using the C3-SC1 leader (^C3-SC1^TECs) are >95% pure, >98% active, and highly synchronous (94% resume transcription in <15 s upon addition of saturating NTPs). We then show that two distinct transcriptional riboswitches remain biochemically active when fused to the C3-SC1 leader. Notably, appending the C3-SC1 leader to these riboswitches reduced basal transcription terminator readthrough but did not reduce the dynamic range of the ligand-induced transcription antitermination response. Overall, these findings establish a procedure for purifying synchronized TECs to homogeneity.

## Results

### Overview of the strategy for purifying synchronized TECs

**Figure 1:**
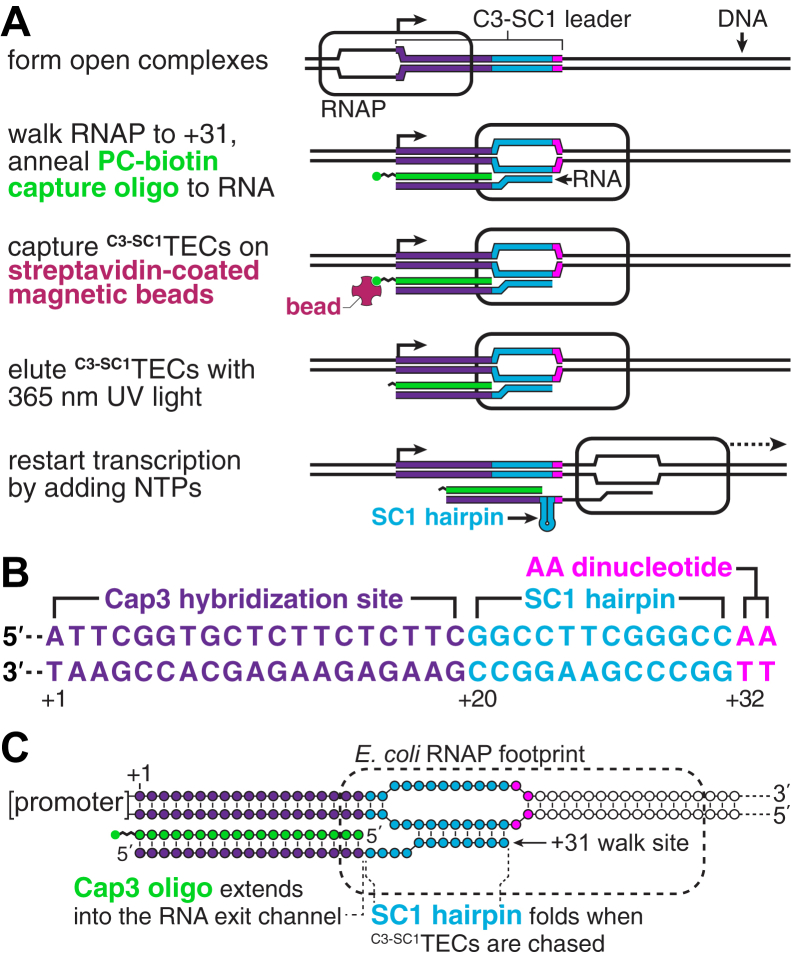
**Overview of the**^**C3-SC1**^**TEC purification strategy.***A*, illustration of the reversible immobilization strategy for isolating ^C3-SC1^TECs. After forming open promoter complexes, single-round transcription is initiated in the absence of ATP to walk RNAP to a synchronization site at C_+31_ of the C3-SC1 leader. During this step, a capture oligonucleotide that contains a photocleavable biotin modification (Cap3_PCBioTEG, Table S1) is annealed to nascent RNA to generate ^C3-SC1^TECs. ^C3-SC1^TECs are then immobilized on streptavidin-coated magnetic beads, and DNA that does not contain a ^C3-SC1^TEC is removed by washing. High-purity ^C3-SC1^TECs can then be eluted by irradiation with 365 nm UV light and used for *in vitro* transcription assays. *B*, annotated sequence of the C3-SC1 leader indicating the location of the Cap3 hybridization site, the SC1 hairpin, and the A_+32_A_+33_ dinucleotide. *C*, architecture of ^C3-SC1^TECs. Approximately two base pairs of the Cap3 oligo-nascent RNA duplex extend into the RNA exit channel of RNAP when the TEC is positioned at C_+31_. C3-SC1, capture sequence 3-structure cassette 1; PC, photocleavable; RNAP, RNA polymerase; TEC, transcription elongation complex.

The purpose of the procedure described below is to isolate synchronized *E. coli* TECs from an *in vitro* transcription reaction. Isolating synchronized TECs requires a strategy for separating TECs from both free DNA and promoter complexes that failed to initiate transcription. One fundamental difference between TECs and nonproductive promoter complexes is that only the former contain nascent RNA. TECs can be immobilized for solid-phase transcription by annealing a biotinylated capture oligonucleotide to nascent RNA (18, 19). We therefore envisioned that the RNA product of an appropriately designed 5′ leader sequence could be used to purify synchronized TECs using a reversible immobilization strategy. In this approach, TECs are walked to a synchronization site and a capture oligonucleotide containing a photocleavable biotin modification (20) is annealed to nascent RNA (Fig. 1*A*). TECs can then be isolated by immobilization on streptavidin-coated magnetic beads and eluted using 365 nm UV light (Fig. 1*A*). The resulting complexes can then be used in downstream applications that require high-purity synchronized TECs.

To facilitate this procedure, we designed a 33 nt 5′ leader sequence, called C3-SC1 (Fig. 1*B*). C3-SC1 is composed of a 30 nt A-less cassette embedded between an initiating A_+1_ nucleotide and an A_+32_A_+33_ dinucleotide. Initiating transcription in the presence of an ApU dinucleotide and in the absence of ATP therefore positions RNAP at C_+31_. The C3-SC1 leader contains two functional elements: Nucleotides +1 to +19, called C3 (capture sequence 3), comprise an oligonucleotide hybridization site that can be used to anneal a functionalized oligo to nascent RNA (Fig. 1, *A* and *B*). Nucleotides +20 to +31 comprise a four GC-pair RNA hairpin with a UUCG tetraloop (21, 22), referred to here as SC1 (structure cassette 1), that sequesters this region of the leader when RNAP is chased from the C_+31_ synchronization site (Fig. 1, *A* and *B*). Several considerations were taken into account when designing the C3 sequence because it functions both as an initial transcribed sequence and as an oligo hybridization site. First, occurrences of the YG dinucleotide, which induces transcription pausing (23, 24, 25, 26, 27, 28), were limited to locations that do not impact escape from the λP_R_ promoter (29). Second, a G_+5_G_+6_ dinucleotide was included because it favors productive initiation from the λP_R_ promoter (29). Third, homopolymeric sequences were limited to a maximum of two nucleotides to minimize the potential for reiterative transcription initiation (30, 31, 32, 33). Fourth, the C3 RNA sequence and its DNA complement are predicted to be unstructured by the ‘fold’ algorithm of the RNAStructure RNA secondary structure prediction software (34). Fifth, the calculated T_m_ of the complementary DNA oligonucleotide (Cap3) is 47.9 °C in our transcription conditions to promote efficient hybridization to nascent RNA at 37 °C.

In the sections below, TECs that have been synchronized using the C3-SC1 leader and contain the Cap3 oligonucleotide are referred to as ^C3-SC1^TECs (Fig. 1*C*). The spatial organization of nucleic acids within ^C3-SC1^TECs was also considered when designing the C3-SC1 leader. The footprint of RNAP on RNA is ∼14 nts (35). Therefore, when RNAP is positioned at the C_+31_ synchronization site, approximately 17 nts of nascent RNA have emerged from the TEC, assuming RNAP does not backtrack (Fig. 1*C*). The 19 nt-long Cap3 oligonucleotide was designed so that approximately 2 bp of the Cap3-nascent RNA hybrid extend into the RNA exit channel of RNAP. This preserves the register of the RNA 3′ end and RNAP active center because nucleic acid structures at the RNA exit channel prevent RNAP from backtracking (36, 37, 38). In addition to functioning as a handle for immobilizing ^C3-SC1^TECs and inhibiting backtracking, the Cap3 oligonucleotide also blocks the C3 sequence from forming base pairs with downstream RNA. Similarly, SC1 forms an RNA hairpin once it emerges from RNAP. In this way, nearly all of the C3-SC1 transcript becomes sequestered in base pairs once RNAP transcribes beyond the synchronization site (Fig. 1*A*).

To be maximally useful, ^C3-SC1^TECs must (1) be homogenous with a 1:1:1 RNAP:DNA:RNA ratio, (2) remain stably associated with DNA during purification, (3) be transcriptionally active, and (4) resume transcription synchronously. In the sections below, we show that ^C3-SC1^TECs isolated using our procedure meet each of these criteria.

### ^C3-SC1^TECs can be immobilized on streptavidin-coated beads

**Figure 2:**
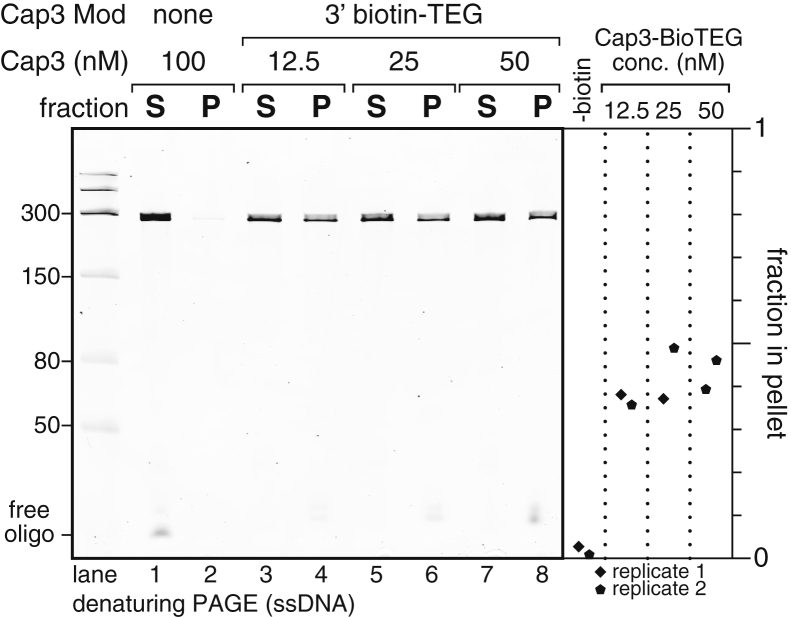
**Immobilization of**^**C3-SC1**^**TECs.** Denaturing PAGE analysis of DNA from ^C3-SC1^TECs that were assembled using variable amounts of the Cap3_NoMod (lanes 1 and 2) or Cap3_BioTEG (lanes 3–8) oligonucleotides (Table S1), immobilized on streptavidin-coated magnetic beads, and partitioned into pellet and supernatant fractions. The plot shows the fraction of DNA that was retained on the streptavidin-coated beads. P, pellet; S, supernatant; TEC, transcription elongation complex.

The purification strategy shown in Figure 1 separates ^C3-SC1^TECs from open promoter complexes and free DNA based on the ability of a biotinylated capture oligonucleotide (Cap3) to hybridize to nascent RNA. To assess the amount of Cap3 needed to efficiently form ^C3-SC1^TECs, we generated ^C3-SC1^TECs with variable concentrations of Cap3 and determined the fraction of template DNA that could be immobilized on streptavidin-coated magnetic beads. Because Cap3 is the only biotinylated molecule in this experiment, a template DNA molecule can only bind to streptavidin-coated beads if it contains a ^C3-SC1^TEC. For this initial experiment, open promoter complexes were formed using a near-saturating excess of *E*. *coli* RNAP relative to 10 nM DNA template (39). Consequently, virtually every template DNA molecule contained an open promoter complex and could potentially yield a ^C3-SC1^TEC if RNAP escaped the promoter and the Cap3 oligonucleotide hybridized to the nascent RNA. The fraction of DNA retained on the magnetic beads therefore reflects the approximate upper bound of ^C3-SC1^TEC capture efficiency in our standard *in vitro* transcription conditions. ^C3-SC1^TECs were formed by initiating transcription in the absence of ATP to walk RNAP to the C_+31_ synchronization site (Fig. 1). This reaction proceeded at 37 °C for 20 min to allow Cap3 to bind to nascent RNA before the reaction was mixed with streptavidin-coated beads. Under these conditions, 36 to 48% of input DNA was captured on the beads when biotinylated Cap3 oligo was present at a concentration of 12.5, 25, or 50 nM (Fig. 2, lanes 3–8). In contrast, >97% of input DNA remained in the supernatant when an unmodified version of Cap3 was included in the transcription reaction (Fig. 2, lanes 1 and 2). It is well-established that a significant fraction of bacterial open promoter complexes become irreversibly trapped in an abortive cycle and fail to initiate transcription (40, 41, 42). Consequently, the efficiency of TEC formation is determined both by the fraction of DNA that contains an open promoter complex and by the fraction of open promoter complexes that escape into productive elongation. We previously observed that transcription initiation from the P_RA1_ promoter, which was used for the experiments in this work, is 50% efficient in the presence of saturating RNAP and NTPs (39). Therefore, the observation that 36 to 48% of input DNA can be captured on streptavidin-coated beads suggests that hybridization of the Cap3 oligonucleotide is efficient and that most unbound DNA did not contain a TEC. Given that Cap3 concentration did not meaningfully change the efficiency of ^C3-SC1^TEC capture, all subsequent experiments in this work were performed using 25 nM Cap3 oligonucleotide.

### Stringent transcription initiation conditions enrich for uniform ^C3-SC1^TECs

**Figure 3:**
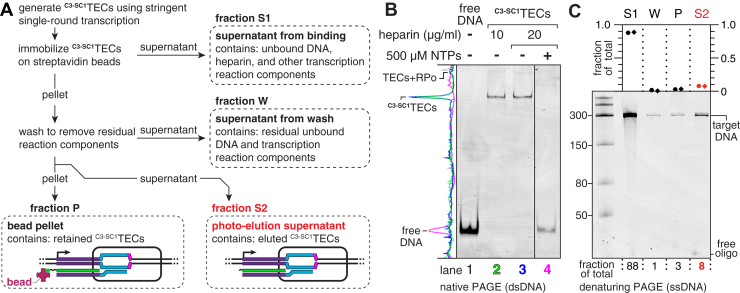
**Purification of homogenous**^**C3-SC1**^**TECs by reversible immobilization.***A*, workflow of the ^C3-SC1^TEC purification procedure. The source of each fraction (S1, W, P, S2) that was collected for the experiment in panel *C* is indicated. *B, EMSA* of ^C3-SC1^TECs that were purified using transcription initiation conditions that favor a 1:1:1 RNAP:DNA:RNA ratio, before and after the resulting complexes were chased by adding NTPs. Lanes 2 and 3 show ^C3-SC1^TECs that were prepared using two concentrations of heparin; three additional independent ^C3-SC1^TEC preparations are shown in Fig. S1*A*. The *solid vertical line* between lanes 3 and 4 indicates a gel splice to remove lanes that were left empty to avoid the potential for NTP contamination in the -NTP samples. The *grayed spike* in the intensity trace for lane 4 corresponds to debris on the gel. *C*, denaturing PAGE analysis of the ^C3-SC1^TEC purification procedure from *A*. The plot shows the fraction of total DNA that was recovered in fractions S1, W, P, and S2. TEC, transcription elongation complex; NTP, nucleoside triphosphate; P, pellet; RPo, open promoter complex; S1, supernatant 1; S2, supernatant 2; W, wash.

^C3-SC1^TECs that were prepared using this stringent transcription initiation procedure contained a fast-migrating major band (>95% of complexes) and a slow-migrating minor band (<5% of complexes) when assessed by EMSA (Fig. 3*B*, lanes 2 and 3). We previously observed a similar migration pattern when assessing the purity of arrested TECs (39); the fast-migrating band corresponded to pure TECs, and the slow-migrating band corresponded to TECs with an associated open promoter complex. To test whether this interpretation applied to ^C3-SC1^TECs, we prepared ^C3-SC1^TECs using a saturating concentration of RNAP and omitted heparin; these conditions favor the formation of new open complexes after RNAP escapes the promoter. As expected, ^C3-SC1^TECs prepared using these conditions contained ∼60% slow-migrating complexes and ∼40% fast-migrating complexes (Fig. S1*B*). We therefore conclude that the fast-migrating band corresponds to pure ^C3-SC1^TECs, and the slow-migrating band corresponds to ^C3-SC1^TECs with an associated open promoter complex. ^C3-SC1^TECs prepared using the stringent transcription initiation conditions described above are therefore >95% homogenous. Importantly, incubating ^C3-SC1^TECs with 500 μM NTPs caused the template DNA to migrate with the same mobility as free DNA, which indicates that RNAP was released from DNA and that the resulting complexes are active (Fig. 3*B*, compare lanes 3 and 4).

The experiments in Figure 2 used a near-saturating concentration of RNAP to assess the efficiency limit of ^C3-SC1^TEC immobilization. However, under these conditions, the exact composition of the immobilized complexes is poorly defined because each DNA molecule can contain both a ^C3-SC1^TEC and an open promoter complex. Ideally, each DNA molecule should contain a maximum of one RNAP so that the RNAP:DNA:RNA ratio is 1:1:1. After evaluating several variations of the ^C3-SC1^TEC purification procedure, we identified stringent transcription initiation conditions that yield >95% pure ^C3-SC1^TECs with a 1:1:1 RNAP:DNA:RNA ratio (Fig. 3 and S1, see Experimental procedures for complete details). First, we used a subsaturating concentration of RNAP when forming open promoter complexes to minimize nonspecific DNA binding. We previously used this strategy when purifying arrested TECs and observed that under these conditions ∼70% of DNA molecules contain an open promoter complex (39); approximately 50% of these complexes are expected to initiate transcription productively in the presence of saturating NTPs under single-round conditions (39), which sets the upper bound for generating ^C3-SC1^TECs to approximately 35% of input DNA molecules. Next, we challenged the open promoter complexes with 20 μg/ml heparin to sequester free RNAP and RNAP that was weakly associated with DNA. Finally, we initiated single-round transcription by simultaneously adding MgCl_2_ and the antibiotic rifampicin to the reaction. This strategy limits the occurrence of abortive cycling during the Cap3 oligonucleotide hybridization and ^C3-SC1^TEC immobilization steps. After assembling and immobilizing ^C3-SC1^TECs using these conditions, the immobilized complexes were washed to remove excess transcription reaction components and eluted by irradiating the sample with 365 nm UV LEDs (∼10 mW/cm^2^) for 5 min (Fig. 3*A*).

The upper bound for ^C3-SC1^TEC yield is 36 to 48% when a near-saturating amount of RNAP is used for transcription (Fig. 2). We anticipated that the stringent conditions required to isolate homogenous complexes would reduce the yield of ^C3-SC1^TECs. To test this, we evaluated the distribution of input DNA across four purification fractions (Fig. 3*A*). As expected, ^C3-SC1^TECs that were purified using the stringent transcription initiation conditions described above contained only 8% of the total input DNA (Fig. 3*C*). Under these conditions, 88% of input DNA did not bind to the streptavidin-coated beads, and 1% of DNA was removed during the wash step (Fig. 3*C*, fractions S1 and W). DNA that was removed in these steps presumably did not contain a ^C3-SC1^TEC. Of the DNA that was immobilized, 3% was not eluted upon irradiation with 365 nm UV light (Fig. 3*C*, fraction P). The total yield of ^C3-SC1^TECs was therefore 11% of total input DNA, and ∼73% of these immobilized complexes were recovered after photoelution (Fig. 3*C*, fractions P and S2). Using a competitor DNA template to sequester free RNAP instead of heparin increased the total yield of ^C3-SC1^TECs to 21%, and 16% of input DNA was recovered in the photoelution supernatant (Fig. S1*D*). However, these conditions reduced the fraction of ^C3-SC1^TECs with a 1:1:1 RNAP:DNA:RNA ratio from >95% to ∼88% (Fig. S1, *B* and *C*). Omitting rifampicin increased the total yield of ^C3-SC1^TECs to 15% (Fig. S1*E*). The low yield of the optimized ^C3-SC1^TEC preparation is therefore due to the combined effects of using a subsaturating RNAP concentration to limit nonspecific DNA binding, using heparin to sequester excess and weakly bound RNAP, and using rifampicin to inhibit abortive cycling while the Cap3 oligonucleotide is annealed to nascent RNA. Nonetheless, this limitation is offset by the high purity and robust activity of the resulting complexes.

### ^C3-SC1^TECs resume transcription synchronously

**Figure 4:**
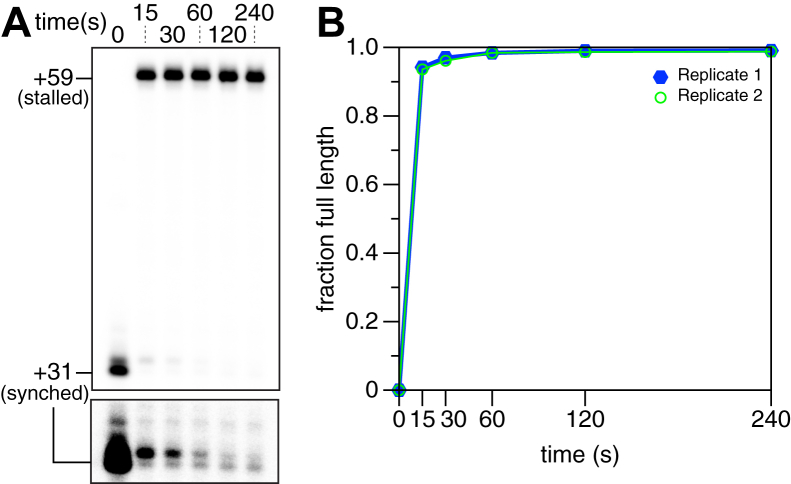
**Analysis of**^**C3-SC1**^**TEC reactivation kinetics.***A*, transcription time course to assess the activity of ^C3-SC1^TECs. RNAP stalls at +59 due to the presence of an etheno-dA modification in the template DNA. The lower cut-out is adjusted to show trace +31 and +32 RNAs in the 15 s to 240 s time points; the entire gel is shown with this darker grayscale setting in Fig. S2*A*. *B*, plot showing the fraction of ^C3-SC1^TECs that transcribed to the +59 stall site at each time point. etheno-dA, 1,N^6^-etheno-2′-deoxyadenosine; RNAP, RNA polymerase; TEC, transcription elongation complex.

The observation that incubating purified ^C3-SC1^TECs with NTPs releases RNAP from DNA suggests that the complexes are transcriptionally active. To assess the nucleic acid composition and reactivation kinetics of ^C3-SC1^TECs more precisely, we prepared ^C3-SC1^TECs that contained ^32^P-labeled RNA and performed a transcription time course. In this experiment, the template DNA contained a 1,N^6^-etheno-2′-deoxyadenosine (etheno-dA) transcription stall site 28 nt downstream of the synchronization site to halt transcription uniformly (43, 44). Before NTPs were added, virtually all ^C3-SC1^TECs contained either a 31 or 32 nt-long RNA (Fig. 4*A*). The presence of a single discrete band at the etheno-dA stall site indicates that the 32 nt RNA is due to transcription 1 nt beyond the C_+31_ synchronization site, likely due to either trace ATP or misincorporation, rather than transcription start site variability (Fig. 4*A*). When transcription was reactivated by adding NTPs to 500 μM, 94% of ^C3-SC1^TECs transcribed to the etheno-dA stall site in <15 s (Fig. 4*B*). Virtually all ^C3-SC1^TECs that did not transcribe to the etheno-dA stall site within 15 s contained a 32 nt-long RNA (Fig. 4*A*). Although this subpopulation resumed transcription slowly, >98% of ^C3-SC1^TECs transcribed to the etheno-dA stall site within 1 minute. Given that purified ^C3-SC1^TECs do not contain free DNA and are >98% active, *in vitro* transcription reactions that use these complexes maintain a quantitative relationship between the template DNA and RNA transcript.

### ^C3-SC1^TECs are compatible with solid-phase transcription reactions

**Figure 5:**
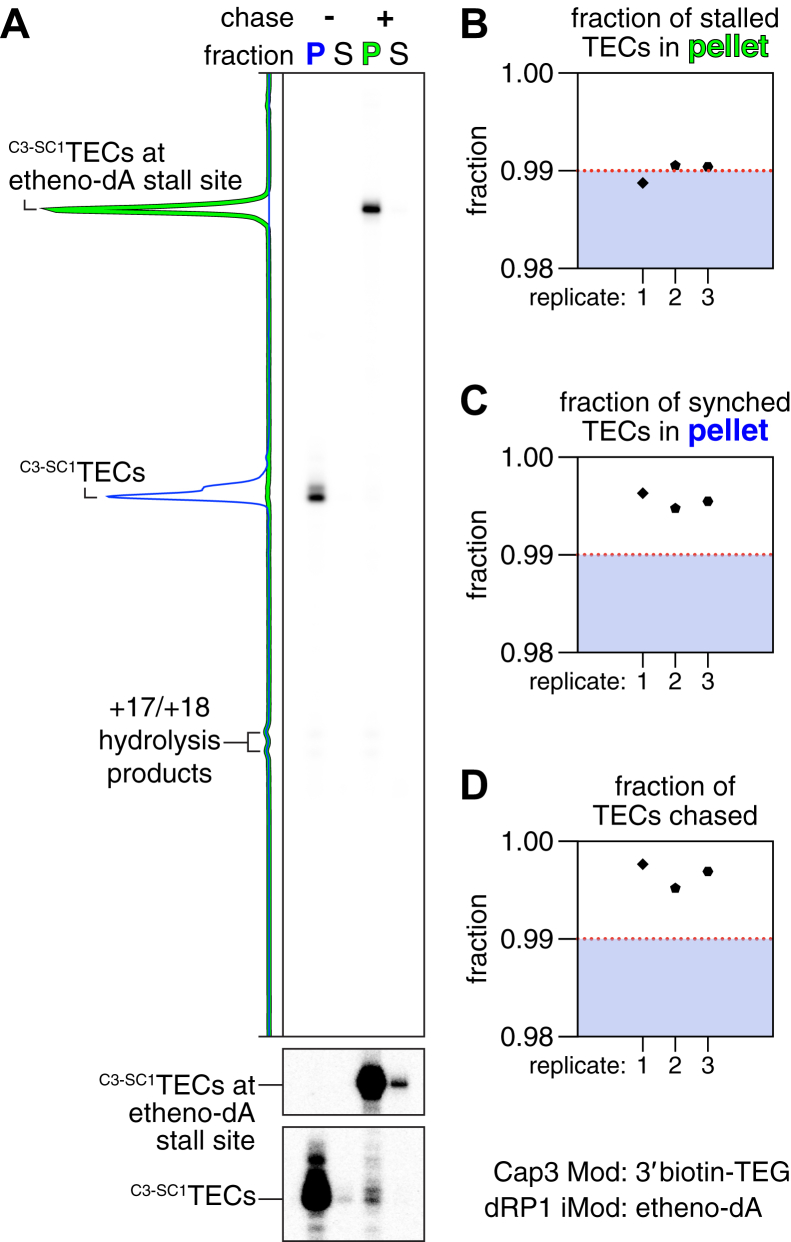
**Solid-phase transcription using**^**C3-SC1**^**TECs.***A*, ^C3-SC1^TECs containing the Cap3_BioTEG oligonucleotide were immobilized on streptavidin-coated magnetic beads and partitioned into pellet and supernatant fractions before and after NTPs were added to reactivate transcription. RNAP stalls at +59 and is retained on DNA due to the presence of an etheno-dA modification in the template DNA. The lower cut-outs are adjusted to show trace synchronized and etheno-dA stalled RNAs in the supernatant fractions; the entire gel is shown with this darker grayscale setting in Fig. S2*B*. The fraction of stalled (*B*) and synchronized (*C*) ^C3-SC1^TECs that were retained in the pellet are shown. *D*, plot showing the fraction of ^C3-SC1^TECs that transcribed to the +59 stall site after NTPs were added. etheno-dA, 1,N^6^-etheno-2′-deoxyadenosine; NTP, nucleoside triphosphate; TEC, transcription elongation complex.

Our analysis of ^C3-SC1^TECs that had been eluted from streptavidin-coated beads established their purity and activity. However, for some applications, it is desirable to perform *in vitro* transcription as a solid-phase reaction (19, 45). To assess whether ^C3-SC1^TECs are compatible with solid-phase transcription, we prepared ‘permanently’ immobilized ^C3-SC1^TECs using a biotinylated Cap3 oligonucleotide that did not contain a photocleavable spacer modification (Cap3_BioTEG, Table S1) and a DNA template that contained an etheno-dA stall site. >99% of ^C3-SC1^TECs remained immobilized both before and after the addition of NTPs, and >99% of ^C3-SC1^TECs transcribed to the etheno-dA stall site (Fig. 5). We therefore conclude that ^C3-SC1^TECs are fully compatible with solid-phase transcription.

We detected trace amounts of transcriptionally inactive 15 to 18 nt-long RNAs (primarily 17 and 18 nts) in both this assay and the transcription time course assay (Fig. S2). With the exception of the 15 nt RNA, these transcripts were retained on the beads and are therefore annealed to the Cap3 oligonucleotide. We therefore conclude that these short transcripts are hydrolysis products of the C3 region of the C3-SC1 transcript that occur during the purification of ^C3-SC1^TECs.

### The C3-SC1 leader is compatible with riboswitch functional assays

**Figure 6:**
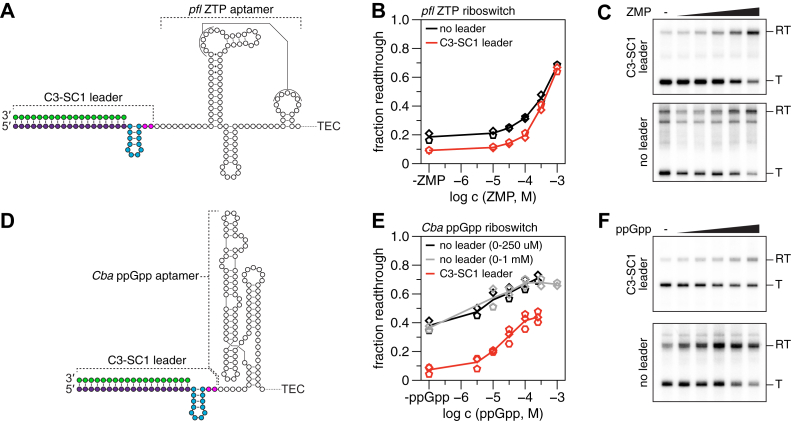
**Transcriptional riboswitch assays using**^**C3-SC1**^**TECs.***A*, illustration of the *Clostridium beijerinckii pfl* ZTP aptamer with the C3-SC1 transcript appended to its 5′ end. *B*, ZMP dose–response curves for the *pfl* ZTP riboswitch with and without the C3-SC1 leader. *C*, representative gels for the analysis in *B*. *D*, illustration of the *Clostridiales bacterium* ppGpp aptamer with the C3-SC1 transcript appended to its 5′ end. *E*, ppGpp dose-response curves for the *Cba* ppGpp riboswitch with and without the C3-SC1 leader. *F*, representative gels for the analysis in *E*. 500 μM NTPs were used for all riboswitch assays. C3-SC1, capture sequence 3-structure cassette 1, *Cba*, *Clostridiales bacterium*; NTP, nucleoside triphosphate; ppGpp, guanosine-3′,5′-bisdiphosphate; RT, readthrough; T, terminated; TEC, transcription elongation complex; ZMP, 5-aminoimidazole-4-carboxamide-1-β-D-ribofuranosyl 5′-monophosphate.

When RNAP transcribes beyond the C_+31_ synchronization site, 31 of 33 nucleotides in the C3-SC1 transcript are sequestered in base pairs with the Cap3 oligonucleotide or in a fast-folding RNA hairpin (Fig. 1). The purpose of this design is to limit interactions between the C3-SC1 transcript and downstream RNA structures that could potentially interfere with RNA function. To determine whether this strategy was successful, we assessed how the C3-SC1 transcript affects the transcription antitermination activity of the *Clostridium beijerinckii pfl* ZTP riboswitch (46) and the *Clostridiales bacterium* (*Cba*) ppGpp (guanosine-3′,5′-bisdiphosphate) riboswitch (47). The C3-SC1 leader was appended to the 5′ end of each riboswitch sequence (Fig. 6, *A* and *D*). We then assessed how the use of ^C3-SC1^TECs in transcription termination assays changes the dose–response curve of each riboswitch relative to control reactions in which the riboswitches contained only the native RNA sequence. In these experiments, the C3-SC1 transcript was considered perturbative to RNA function if the dose–response observed when transcription was performed using ^C3-SC1^TECs deviated from the dose–response observed for native riboswitches in any way. When transcription was performed using ^C3-SC1^TECs, the basal amount of terminator readthrough observed in the absence of ligand was reduced from 19% to 9% for the *pfl* riboswitch and from 38% to 7% for the *Cba* riboswitch (Fig. 6, *B* and *E*). The C3-SC1 transcript did not meaningfully reduce the amount of transcription antitermination observed for the *pfl* ZTP riboswitch at the highest ZMP (5-aminoimidazole-4-carboxamide-1-β-D-ribofuranosyl 5′-monophosphate) concentration tested (1 mM ZMP) and consequently increased fold-change from 3.6 to 7.1 over this concentration range (Fig. 6, *B* and *C*). In contrast, the C3-SC1 transcript decreased the amount of transcription terminator readthrough observed for the *Cba* ppGpp riboswitch at all concentrations tested (Fig. 6, *E* and *F*). Although the difference between terminator readthrough without ppGpp and at saturating ppGpp remained approximately constant, (37% for ^C3-SC1^TECs, 32% for the native riboswitch), the reduction in ligand-independent terminator readthrough observed for ^C3-SC1^TECs increased fold-change from 1.9 to 6.0. The observation that the C3-SC1 transcript increases the efficiency of both riboswitch terminators suggests that it increases the rate of terminator folding, presumably by destabilizing the apo riboswitch aptamers. Nonetheless, the observation that the dynamic range of the transcription antitermination response remained constant for the *Cba* riboswitch and increased for the ZTP riboswitch suggests that the C3-SC1 transcript did not cause either aptamer to misfold catastrophically. Given these findings, we conclude that transcription using ^C3-SC1^TECs is generally perturbative to RNA function but that these perturbations do not necessarily interfere with function.

## Discussion

We have developed a procedure for isolating synchronized TECs from an *in vitro* transcription reaction using a photoreversible immobilization strategy. Our approach uses a custom 5′ leader sequence called C3-SC1 to generate synchronized TECs that can be immobilized, washed, and recovered by 365 nm UV irradiation (Fig. 1). TECs isolated using this procedure (^C3-SC1^TECs) are >95% pure, >98% active, compatible with solid-phase transcription, and can be used in biochemical assays for RNA function. Importantly, the purification of ^C3-SC1^TECs establishes a 1:1:1 ratio between RNAP, template DNA, and the nascent RNA transcript, and the high activity of the resulting complexes maintains this ratio when transcription is resumed. These properties make ^C3-SC1^TECs a potentially powerful tool for developing biochemical and biophysical assays that require high-performance, quantitative bacterial *in vitro* transcription.

Our procedure for isolating ^C3-SC1^TECs is optimized for purity and activity but has three primary limitations. First, initiating transcription using stringent conditions that maximize the purity of ^C3-SC1^TECs reduces the fraction of total input DNA that can be immobilized due to the presence of a ^C3-SC1^TEC from 36 to 48% (when transcription is performed with saturating RNAP and without heparin or rifampicin) to 11%. When ^C3-SC1^TECs are recovered from streptavidin-coated magnetic beads by photoelution, the yield is 8% of total input DNA. Challenging open promoter complexes with a competitor DNA template instead of heparin doubled the yield but reduced the purity of the ^C3-SC1^TECs from >95% to ∼88% and is therefore not ideal. Second, excess Cap3 oligonucleotide cannot be removed from the ^C3-SC1^TEC preparation easily because it contains the attachment chemistry needed to pull down the target complexes. Although we cannot definitively state that free Cap3 oligonucleotide was nonperturbative to riboswitch function, both riboswitches assessed in this work remained functional when transcribed with ^C3-SC1^TECs (Fig. 6). However, the effect of free Cap3 oligonucleotide on downstream biochemical assays will likely be application-dependent. Third, the procedure that we have described is not necessarily compatible with all bacterial promoters. In the experiments described above, we used a custom σ^70^ promoter that is primarily derived from the λP_R_ promoter (39), which generates long-lived, heparin-resistant open complexes (48). Promoters that generate short-lived, heparin-sensitive open complexes will not be compatible with our procedure. Given these limitations, ^C3-SC1^TECs will be most useful for experiments that demand the use of pure, synchronous TECs to transcribe complex RNA molecules, but which do not require the use of a specific promoter or obtaining concentrated transcription complexes. In this way, the procedure we have described complements existing scaffold-based strategies for assembling high-purity synchronized TECs from DNA and RNA oligonucleotides (49, 50). Nucleic acid scaffolds can be used prepare high-purity synchronized TECs at micromolar concentrations but can only be used to synthesize long transcripts if the scaffold DNA is ligated to a second template DNA molecule (50). In contrast, ^C3-SC1^TECs can be assembled on long or complex template DNA molecules because they are generated by promoter-directed transcription initiation using standardized sequences.

Because the ability of leader transcripts to perturb RNA function is well-established (51), the C3-SC1 leader was designed to minimize base-pairing interactions with downstream RNA. Nonetheless, the C3-SC1 transcript perturbed the transcription antitermination activity of ZTP- and ppGpp-sensing transcriptional riboswitches (Fig. 6). Although this outcome was not surprising, its nature was unexpected: transcription using ^C3-SC1^TECs reduced the probability that RNAP bypasses the transcription termination site in the absence of ligand but did not interfere with the ability of either riboswitch to antiterminate transcription in the presence of ligand. In effect, ^C3-SC1^TECs improved the biochemical activity of both riboswitch systems by increasing the fold-change of the switches. One possible explanation for this effect is that the C3-SC1 transcript destabilizes the apo ZTP and ppGpp aptamers so that terminator hairpin folding is more efficient but does not cause the aptamers to catastrophically misfold. Regardless of the mechanistic origin of this effect, its occurrence underscores the importance of evaluating how the C3-SC1 transcript impacts RNA function when using ^C3-SC1^TECs. Given that the C3-SC1 transcript did not interfere with the function of either of the structurally distinct riboswitches assessed in this work, it is likely that ^C3-SC1^TECs will be compatible with many RNA targets. Furthermore, ^C3-SC1^TECs may be useful for manipulating the activity of riboswitch RNAs *in vitro* if the functional enhancements that we observed for the ZTP and ppGpp riboswitches are generalizable to other systems.

Like bacterial RNAPs, transcription elongation by bacteriophage (52, 53), viral (54, 55, 56, 57), and eukaryotic (50, 58, 59) RNAPs can be controlled by withholding one or more NTPs from an *in vitro* transcription reaction. It is therefore likely that the system for isolating synchronized *E*. *coli* TECs described here can be adapted for other RNAPs. Several considerations should be accounted for when adapting or reimplementing the C3-SC1 leader for other RNAPs. First, only RNAPs that are capable of initiating transcription at an A-less cassette will potentially be compatible with the C3-SC1 leader. C3-SC1 was implemented using an A-less cassette to permit a UUCG tetraloop in the SC1 hairpin. If an A-less cassette cannot be used, it may be possible to design a similar leader with a U-less cassette since this would permit an SC1-like hairpin that is GC-rich but contains an alternate tetraloop motif such as GNRA. The use of G-less or C-less cassettes is disadvantageous because it precludes using a GC-rich hairpin to sequester leader nucleotides. However, it may be possible to overcome this limitation by using thermostability enhancing nucleotides like 2-aminoadenosine-5′-triphosphate when walking RNAP to the synchronization site. Second, the C3 sequence was designed to facilitate efficient transcription initiation by *E*. *coli* RNAP. Elements within this sequence, such as the initiating A_+1_T_+2_ dinucleotide, may not be optimal for other RNAPs but can be adapted as needed. For example, the oligonucleotide hybridization site is predicted to remain unstructured when the A_+1_T_+2_ dinucleotide of C3 is substituted with the G_+1_G_+2_ dinucleotide that is preferred by T7 RNAP. Third, the nucleic acid architecture of ^C3-SC1^TECs was designed to prevent backtracking by positioning two base pairs of the Cap3 oligonucleotide-nascent RNA duplex in the RNA exit channel of *E*. *coli* RNAP. This feature can likely be tailored for most RNAPs by placing the 5′ end of the capture oligonucleotide at a position that inhibits backtracking as long as the T_m_ remains sufficient for efficient and stable hybridization and the capture oligo remains unstructured. Finally, the procedure for isolating synchronized TECs requires the use of single-round transcription. This can typically be accomplished by using heparin to sequester free RNAP before transcription is initiated (60), and Passalacqua *et al*. have recently described a single-round transcription strategy in which promoter DNA is enzymatically cleaved after walking T7 RNAP to a synchronization site (53). In general, the design considerations that we have described above in the section *Overview of the strategy for purifying synchronized TECs* will be a useful starting point when adapting the strategies presented in this work for other RNAP enzymes.

The system we have described establishes a means for performing *E*. *coli in vitro* transcription reactions in which virtually every template DNA molecule yields one RNA molecule. In principle, this capability could facilitate the development of high-throughput sequencing assays in which template DNA molecules are partitioned by the biochemical activity of nascent RNA. In this experiment format, nascent RNA activity would be reflected by the distribution of template DNA across fractions, which can be determined by DNA sequencing. This general strategy is potentially useful for circumventing technical biases from enzymatic processing steps that are typically required for RNA sequencing experiments and for developing experiments in which sequence information can be recovered from DNA even if RNA is destroyed during the assay. ^C3-SC1^TECs may also be useful for developing assays that require the quantitative functionalization of transcription complexes with a modified oligo because every ^C3-SC1^TEC is guaranteed to contain the Cap3 oligonucleotide. Importantly, ^C3-SC1^TECs are fully compatible with both solution-based and solid-phase transcription experiments (Figs. 4 and 5). The first implementation of solid-phase transcription by Kashlev *et al*. (45) involved immobilizing hexahistidine-tagged RNAP on Ni^2+^-chelating agarose beads. Following its conception, solid-phase transcription technology has been extended to include diverse immobilization strategies in which transcription complexes are fixed to a surface by chemically modified template DNA (49), nascent RNA (6), and DNA handles (3) or oligonucleotides (18) that are annealed to nascent RNA (as is the case for the system described here). However, previous systems for solid-phase transcription using promoter-initiated TECs have not been optimized to both establish and maintain a 1:1 ratio between DNA and RNA molecules. The procedure we have described is therefore differentiated from established methods for solid-phase transcription because the C3-SC1 leader is engineered to facilitate quantitative *in vitro* transcription using precisely defined TECs. Consequently, ^C3-SC1^TECs can be used in combination with precision transcription roadblocking methods (43) to perform solid-phase transcription reactions in which >99% of immobilized complexes are transcriptionally active and remain immobilized during the assay (Fig. 5). In sum, the central advance of this work is the establishment of a versatile, standardized system for quantitative *E*. *coli in vitro* transcription. We anticipate that this technology will enable the development of new experimental methods that would not otherwise be possible.

## Experimental procedures

### Oligonucleotides

All oligonucleotides were purchased from Integrated DNA Technologies. A detailed description of all oligonucleotides including sequence, modifications, and purifications is presented in Table S1. Out of an abundance of caution, oligonucleotides that contained a PC spacer modification were handled under low intensity 592 nm light from SimpleColor Amber LEDs (Waveform Lighting) set to 40% intensity using a FilmGrade Flicker-Free LED Dimmer (Waveform Lighting) and stored as single-use aliquots.

### Proteins

Vent (exo-) DNA polymerase, Q5 High-Fidelity DNA Polymerase, *Sulfolobus* DNA Polymerase IV, and *E*. *coli* RNAP holoenzyme were purchased from New England Biolabs. NusA was a gift from J. Roberts (Cornell University).

### DNA template sequences

DNA template sequences are available in Table S2. Fully annotated versions of DNA templates 1 (https://benchling.com/s/seq-FUAWrFQwiHR3aJmAUExl), 2 (https://benchling.com/s/seq-3hXcRKbqeVo9u4Hhl2oI), 3 (https://benchling.com/s/seq-wIQ0VHiDjt1ahpC9ELjD), 4 (https://benchling.com/s/seq-XFA473UmWVvfLaubWzXa), 5 (https://benchling.com/s/seq-2QwZ9VceV9l3uarzKCKT), and 6 (https://benchling.com/s/seq-LI54uN63QQ9QyAAJXIAi) are available at Benchling.

### DNA template preparation

Table S3 provides details for the oligonucleotides and processing steps used for every DNA template preparation used in this work. Unmodified DNA templates were PCR amplified from plasmid DNA using Vent (exo-) DNA polymerase and purified by agarose gel extraction exactly as described previously (39). DNA templates that contained an internal etheno-dA transcription stall site were PCR amplified from an unmodified linear dsDNA using Q5 High-Fidelity DNA polymerase; the unmodified linear dsDNA that was used as a template for these reactions was PCR amplified from plasmid DNA using Vent (exo-) DNA polymerase and purified by agarose gel extraction. After PCR amplification, DNA containing an internal etheno-dA modification was purified using a QIAquick PCR Purification Kit (Qiagen), processed by translesion DNA synthesis using a mixture of *Sulfolobus* DNA Polymerase IV and Vent (exo-) DNA polymerase, and purified a second time using a QIAquick PCR Purification Kit; these steps were performed exactly as described previously (39). A step-by-step protocol describing this procedure is available (61).

### Preparation of streptavidin-coated magnetic beads

Five microliters of 10 mg/ml Dynabeads MyOne Streptavidin C1 beads (Invitrogen) per 25 μl sample volume were prepared in bulk exactly as described previously (39). The resulting beads were resuspended at a concentration of ∼2 μg/μl in Buffer TX (1X transcription buffer [20 mM Tris-HCl (pH 8.0), 50 mM KCl, 1 mM DTT, 0.1 mM EDTA (pH 8.0)] supplemented with 0.1% Triton X-100), split into in 25 μl aliquots, and stored on ice until use.

### Capture oligo dose assays

For the capture oligo dose assay in Figure 2, 25 μl *in vitro* transcription reactions containing 1X transcription buffer, 0.1 mg/ml BSA, 10 mM MgCl_2_, 100 μM ApU dinucleotide (Dharmacon), 1X UGC Start NTPs (25 μM UTP, 25 μM GTP, 25 μM CTP, all NTP mixtures were prepared using High Purity rNTP solutions [Cytiva]), 10 nM DNA Template 1 (Tables S2 and S3), 0.024 U/μl *E coli* RNAP holoenzyme, and Cap3 oligonucleotide (either 12.5, 25, or 50 nM Cap3_BioTEG or 100 μM Cap3_NoMod, Table S1) were prepared in a 1.7 ml microcentrifuge tube on ice; at this point, the total reaction volume was 22.5 μl due to the omission of 10X (100 μg/ml) rifampicin.

Transcription reactions were placed in a dry bath set to 37 °C for 20 min to walk RNAP to C_+31_ of the C3-SC1 leader. After 20 min, rifampicin was added to a final concentration of 10 μg/ml, and the sample was incubated at 37 °C for five additional minutes to block new transcription initiation. Pre-equilibrated, room-temperature streptavidin-coated magnetic beads were placed on a magnetic stand, and storage buffer was removed. The streptavidin-coated magnetic beads were resuspended with the transcription reaction by pipetting and incubated at room temperature with rotation for 15 min. After 15 min, the bead binding mixture was spun briefly in a Labnet Prism mini centrifuge (Labnet International) by quickly flicking the switch on and off so that liquid was removed from the tube cap. The sample was placed on a magnetic stand for 1 min, the supernatant was transferred to a 1.7 ml microcentrifuge tube containing 125 μl stop solution [0.6 M Tris-HCl (pH 8.0), 12 mM EDTA (pH 8.0)], and the pellet was resuspended in 25 μl of 95% deionized formamide and 10 mM EDTA, heated at 95 °C for 5 min, placed on a magnetic stand for 1 min, and the supernatant was collected and mixed with 125 μl of stop solution. The samples were then processed by phenol:chloroform extraction and ethanol precipitation and fractionated by denaturing Urea-PAGE as described below in the section *Collection, processing, and denaturing PAGE of* ^*C3-SC1*^*TEC purification fractions*.

### Preparation of ^C3-SC1^TECs

The transcription conditions used for the preparation of ^C3-SC1^TECs were selected to enrich for active transcription complexes. First, a subsaturating amount of *E. coli* RNAP (determined previously (39)) was used to minimize nonspecific DNA binding during open complex formation. Second, open promoter complexes were challenged with heparin (Sigma-Aldrich, catalog # H5515) to sequester free RNAP and enrich for heparin-resistant open complexes. Third, transcription was limited to a single-round using rifampicin so that transcription was not actively occurring during the Cap3 oligonucleotide hybridization and streptavidin-coated bead binding steps. Below, the final validated procedure is detailed first, and other variations of the procedure that were performed during protocol development are then described with reference to the figures in which they were used.

Twenty-five microliter *in vitro* transcription reactions containing 1X transcription buffer, 0.1 mg/ml BSA, 100 μM ApU dinucleotide, 1X UGC Start NTPs (25 μM UTP, 25 μM GTP, 25 μM CTP), 10 nM template DNA, 0.016 U/μl *E coli* RNAP holoenzyme, and 25 nM Cap3_PCBioTEG oligonucleotide (Table S1) were prepared in a 1.7 ml microcentrifuge tube on ice; at this point, the total reaction volume was 20 μl due to the omission of 10X (200 μg/ml) heparin and 10X start solution (100 mM MgCl_2_, 100 μg/ml rifampicin). The Cap3_PCBioTEG oligonucleotide was added to the reaction last under 592 nm amber light, and all sample handling until the 365 nm UV irradiation step was performed under 592 nm amber light. When preparing radiolabeled ^C3-SC1^TECs, 0.2 μCi/μl [α-^32^P]UTP (PerkinElmer) was included in the transcription reaction.

Transcription reactions were placed in a dry bath set to 37 °C for 20 min to form open promoter complexes. After 20 min, 2.5 μl of 200 μg/ml heparin per sample volume was added to the reaction, and the sample was mixed by pipetting and incubated at 37 °C for 5 min to sequester free RNAP and enrich for heparin-resistant open promoter complexes; the final concentration of heparin was 20 μg/ml. After 5 min, 2.5 μl of room temperature 10X start solution per sample volume was added to the transcription reaction for a final concentration of 10 mM MgCl_2_ and 10 μg/ml rifampicin. The transcription reaction was mixed by pipetting and incubated at 37 °C for 20 min to walk RNAP to the C_+31_ synchronization site and hybridize the Cap3 oligonucleotide. At this time, tubes containing 25 μl of ∼2 μg/μl pre-equilibrated streptavidin-coated magnetic beads and Buffer TMW (1X transcription buffer, 10 mM MgCl_2_, and 0.05% Tween-20) were placed at room temperature.

After ∼18 min, the magnetic beads were placed on a magnetic stand, and storage buffer was removed. After 20 min, the beads were resuspended with the transcription reaction by pipetting and incubated in the dark at room temperature with rotation for 15 min. After 15 min, the bead binding mixture was spun briefly in a Labnet Prism mini centrifuge by quickly flicking the switch on and off so that liquid was removed from the tube cap, but the speed of the mini centrifuge remained as low as possible. The sample was placed on a magnetic stand for 1 min, and the supernatant was removed; this supernatant, which contained any transcription reaction components that did not bind the beads, is referred to as fraction S1. The 1.7 ml tube containing the sample was removed from the magnetic stand, and the beads were gently resuspended in either 250 μl (for single samples) or 1 ml (for bulk samples) of room temperature Buffer TMW and incubated in the dark at room temperature with rotation for 5 min. The sample was placed on a magnetic stand for 1 min, and the supernatant was removed; this supernatant, which contains residual transcription reaction components, is referred to as fraction W. In all experiments except the riboswitch functional assays described below, the beads were gently resuspended in 25 μl of Buffer TMW per sample volume by pipetting so that the bead concentration was ∼2 μg/μl, placed in a custom-built 365 nm UV LED irradiator for 1.7 ml microcentrifuge tubes, and exposed to ∼10 mW/cm^2^ 365 nm UV light from four directions for 5 min (39); all sample handling was performed under room light after this step was complete. After UV irradiation, the sample was returned to the magnetic stand for 1 min, and the supernatant was collected. The bead pellet, which contains any ^C3-SC1^TECs that were not eluted by 365 nm UV irradiation, is referred to as fraction P. The collected supernatant, which contains eluted ^C3-SC1^TECs, is referred to as fraction S2.

Several variations of this protocol were performed. In addition to preparing ^C3-SC1^TECs with 20 μg/ml heparin, ^C3-SC1^TECs were also prepared with 10 μg/ml and 15 μg/ml heparin without any detectable difference (Figs. 3 and S1*A*). In preliminary experiments, open promoter complexes were incubated with 15, 22.5, or 30 nM competitor DNA containing an etheno-dA lesion for 20 min in place of heparin (Fig. S1, *B*–*D*). In control experiments ^C3-SC1^TECs were prepared using 5 nM DNA template, 0.024 U/μl *E coli* RNAP holoenzyme, and no heparin or competitor DNA (Fig. S1*B*) and without rifampicin (Fig. S1*E*).

### Analysis of ^C3-SC1^TECs by EMSA

EMSAs were performed exactly as described previously (39) by fractionating samples with a 0.5X Tris-borate-EDTA 5% polyacrylamide gel prepared for a Mini-PROTEAN Tetra Vertical Electrophoresis Cell (Bio-Rad) using ProtoGel acrylamide (National Diagnostics). To assess purified ^C3-SC1^TECs, 15 μl of a 25 μl sample was mixed with 3 μl of 6X Native DNA Loading Dye (30% (v/v) glycerol, 10 mM Tris-HCl (pH 8.0), 0.01% (w/v) bromophenol blue), and loaded onto the gel. To assess ^C3-SC1^TECs after reactivating transcription, a 25 μl sample was mixed with 0.5 μl of 500 μg/ml rifampicin, incubated at 37 °C for 2 min, mixed with 0.5 μl of 25 mM NTPs, and incubated at 37 °C for 2 min; 15.6 μl of the 26 μl reaction was mixed with 3 μl of 6X Native DNA Loading Dye and loaded onto the gel. After the gel had run for ∼2 h and 45 min at 45 V, the gel was transferred to a plastic dish containing 1X SYBR Gold (Invitrogen) in 0.5X Tris-borate-EDTA, stained for 10 min with rocking, and scanned on a Sapphire Biomolecular imager using the 488 nm/518BP22 setting.

### Collection, processing, and denaturing PAGE of ^C3-SC1^TEC purification fractions

^C3-SC1^TEC purification fractions were collected and processed as follows: fraction S1 was mixed with 125 μl of stop solution. Fraction W was mixed with 5 μl of 0.5 M EDTA (pH 8.0). To recover immobilized nucleic acids from fraction P, the bead pellet was resuspended in 25 μl of 95% deionized formamide and 10 mM EDTA, heated at 95 °C for 5 min, placed on a magnetic stand for 1 min, and the supernatant was collected and mixed with 125 μl of stop solution. Fraction S2 was mixed with 125 μl of stop solution. The fractions were extracted by adding an equal volume of Tris (pH 8) buffered phenol:chloroform:isoamyl alcohol (25:24:1, v/v), mixing by vortexing and inversion, and centrifuging at 18,500*g* and 4 °C for 5 min. The aqueous phase was collected and transferred to a new tube. Nucleic acids were precipitated by adding 0.1 sample volumes of 3 M sodium acetate (pH 5.5), three sample volumes of 100% ethanol, and 1 μl of GlycoBlue Coprecipitant (Invitrogen), and chilling at −70 °C for 30 min. The samples were centrifuged at 18,500*g* and 4 °C for 30 min, the supernatant was removed, the samples were centrifuged again briefly to pull down residual ethanol, and residual ethanol was removed. The pellet was dissolved in 16 μl of formamide loading dye (90% (v/v) deionized formamide, 1X transcription buffer, 0.01% (w/v) bromophenol blue), heated at 95 °C for 5 min, and snap-cooled on ice for 2 min. The samples were then assessed by Urea-PAGE using an 8% gel prepared with the SequaGel UreaGel 19:1 Denaturing Gel System (National Diagnostics) for a Mini-PROTEAN Tetra Vertical Electrophoresis Cell exactly as described previously (39).

### Transcription kinetics assay

Seven sample volumes of ^C3-SC1^TECs containing [α-^32^P]UTP-labeled RNA were prepared in bulk using DNA template 2 (Tables S2 and S3) as described in the section *Preparation of* ^*C3-SC1*^*TECs*. The resulting 175 μl pooled sample was mixed with 3.5 μl of 500 μg/ml rifampicin and incubated at 37 °C for 2 min. To take a zero time point, 25 μl of the pooled sample was removed, mixed with 125 μl of stop solution, and kept on ice. The remaining 150 μl pooled sample was mixed with 3 μl of 25 mM NTPs for a final NTP concentration of ∼500 μM NTPs and incubated at 37 °C. At each time point (15 s, 30 s, 1 min, 2 min, and 4 min), a 25 μl sample volume was removed from the pooled sample, mixed with 125 μl of stop solution, and transferred to ice. The samples were processed as described below in the section *Purification, sequencing gel electrophoresis, and detection of radiolabeled RNA*.

### Immobilized ^C3-SC1^TEC activity assay

Immobilized ^C3-SC1^TECs containing [α-^32^P]UTP-labeled RNA were prepared in bulk using DNA template 2 (Tables S2 and S3) as described in the section *Preparation of* ^*C3-SC1*^*TECs* except the Cap3_BioTEG oligonucleotide (Table S1) was used instead of Cap3_PCBioTEG, and the photoelution step was omitted. Fifty microliters of immobilized ^C3-SC1^TECs were mixed with 1 μl 500 μg/ml rifampicin and incubated at 37 °C for 2 min. Twenty-five microliters of the sample was transferred to a separate 1.7 ml microcentrifuge tube and placed on a magnetic stand for 1 min. The supernatant was transferred to 125 μl of stop solution, and the beads were resuspended in 25 μl of 95% deionized formamide and 10 mM EDTA. The remaining 25 μl of the sample was mixed with 0.5 μl of 25 mM NTPs for a final concentration of ∼500 μM NTPs, incubated at 37 °C for 1 min, and placed on a magnetic stand for 1 min. The supernatant was transferred to 125 μl of stop solution, and the beads were resuspended in 25 μl of 95% deionized formamide and 10 mM EDTA. To recover immobilized nucleic acids, the pellet fractions were heated at 95 °C for 5 min, placed on a magnetic stand for 1 min, and the supernatant was collected and mixed with 125 μl of stop solution. The samples were processed as described below in the section *Purification, sequencing gel electrophoresis, and detection of radiolabeled RNA*.

### Transcription termination assays

Transcription termination assays using ^C3-SC1^TECs were performed as follows: ^C3-SC1^TECs containing [α-^32^P]UTP-labeled RNA were prepared in bulk using either DNA template 1 or 4 (Tables S2 and S3) as described above in the section *Preparation of* ^*C3-SC1*^*TECs*, except that ^C3-SC1^TECs were eluted into either 19.5 μl per sample volume of elution buffer Z (1.28X transcription buffer, 12.82 mM MgCl_2_, 0.05% Tween 20) for ZTP riboswitch assays or 18.75 μl per sample volume of elution buffer G (1.33X transcription buffer, 13.33 mM MgCl_2_, 0.07% Tween 20, 0.67 μM NusA) for ppGpp riboswitch assays. For ZTP riboswitch assays, eluted ^C3-SC1^TECs were prewarmed at 37 °C for 2 min before 19.5 μl of ^C3-SC1^TECs were mixed with 5.5 μl of prewarmed Chase Mix Z (2.5 μl 5 mM NTPs, 2.5 μl 100 μg/ml rifampicin, 0.5 μl of ZMP (Sigma Aldrich) in DMSO at 50X final ZMP concentration), incubated at 37 °C for 5 min, and mixed with 125 μl of stop solution. For ppGpp riboswitch assays, eluted ^C3-SC1^TECs were prewarmed at 37 °C for 2 min before 18.75 μl of ^C3-SC1^TECs were mixed with 6.25 μl of prewarmed Chase Mix G (2.5 μl 5 mM NTPs, 2.5 μl 100 μg/ml rifampicin, 1.25 μl of ppGpp (Jena Bioscience) in nuclease-free water at 20X final ppGpp concentration), incubated at 37 °C for 5 min, and mixed with 125 μl of stop solution.

Transcription termination assays for leader-less riboswitches were performed as follows: 25 μl transcription reactions containing 1X transcription buffer, 0.1 mg/ml BSA, 10 mM MgCl_2_, 1X AGU Start NTPs (2.5 μM ATP, 2.5 μM GTP, 1.5 μM UTP), 0.2 μCi/μl [α-^32^P]UTP, 5 nM DNA template 3 or 5 (Tables S2 and S3), and 0.016 U/μl *E coli* RNAP were prepared in a 1.7 ml microcentrifuge tube on ice; at this point, the total reaction volume was 19.5 μl per sample volume for ZTP riboswitch assays and 18.75 μl per sample volume for ppGpp riboswitch assays due to the omission of Chase Mix Z and Chase Mix G, respectively. Transcription reactions were incubated at 37 °C for 10 min to walk RNAP to the first C in the riboswitch sequence (+15 for the ZTP riboswitch and +17 for the ppGpp riboswitch). For ZTP riboswitch assays, 19.5 μl of the master mix was mixed with 5.5 μl of prewarmed Chase Mix Z, incubated at 37 °C for 5 min, and mixed with 125 μl of stop solution. For ppGpp riboswitch assays, 18.75 μl of the master mix was mixed with 6.25 μl of prewarmed Chase Mix G, incubated at 37 °C for 5 min, and mixed with 125 μl of stop solution. The samples were processed as described below in the section *Purification, sequencing gel electrophoresis, and detection of radiolabeled RNA*.

### Purification, sequencing gel electrophoresis, and detection of radiolabeled RNA

Radiolabeled RNA from *in vitro* transcription reactions was processed and analyzed exactly as described previously (43). Briefly, 150 μl samples (25 μl sample +125 μl Stop Solution) were mixed with an equal volume (150 μl) of Tris (pH 8) buffered phenol:chloroform:isoamyl alcohol (25:24:1, v/v), mixed by vortexing and inversion, and centrifuged at 18,500*g* and 4 °C for 5 min. The aqueous phase was collected and transferred to a new tube. Nucleic acids were precipitated by adding three sample volumes (450 μl) of 100% ethanol and 1 or 1.2 μl of GlycoBlue Coprecipitant and chilling at −70 °C for at least 30 min. The samples were centrifuged at 18,500*g* and 4 °C for 30 min, the supernatant was removed, the samples were centrifuged again briefly to pull down residual ethanol, and residual ethanol was removed. The pellets were dissolved in 6.5 μl of formamide loading dye, denatured by heating at 95 °C for 5 min, loaded on a prewarmed 7.5 M urea, 12% polyacrylamide, 35 × 43 cm, 0.4 mm thick sequencing gel in a Model S2 Sequencer Apparatus, and run at 1400 V for 2.5 to 3 h. The resulting gel was exposed to a storage phosphor screen for 12 to 16 h, and the storage phosphor screen was scanned using an Amersham Typhoon IP Biomolecular Imager (Cytiva).

### Quantification

Quantification of band intensity was performed by analyzing gel image TIFF files using ImageJ 1.53k exactly as described previously (39). The distribution of DNA between pellet and supernatant fractions (Fig. 2), the purity of ^C3-SC1^TECs (Fig. 3*B*), and the distribution of input DNA between S1, W, P, and S2 fractions (Fig. 3*C*) were calculated exactly as described previously for roadblocked TECs (39).

In experiments that used radiolabeled ^C3-SC1^TECs, all [α-^32^P]UTP was removed before the ^C3-SC1^TECs were chased; therefore, all RNA species were uniformly labeled, and no normalization was applied. In functional assays using leader-less riboswitches, transcripts were considered end-labeled, as described previously (9), due to the high probability of [α-^32^P]UTP incorporation during the initial walk (∼4.25% per U nucleotide) and the low probability of [α-^32^P]UTP incorporation during the chase (∼0.013% per U nucleotide), and no normalization was applied. In Figure 4*B*, fraction full length was calculated by dividing the band intensity of etheno-dA stalled transcripts by the total band intensity of stalled and synched transcripts. In Figure 5, *B* and *C*, the fraction of synched/stalled TECs that were retained on beads was calculated by dividing the band intensity of RNA in the pellet fraction by the sum of pellet and supernatant band intensities for each indicated complex. In Figure 5*D*, the fraction of TECs chased was calculated by dividing the sum of the pellet and supernatant band intensities of etheno-dA stalled transcripts by the total band intensity of stalled and synched transcripts in both fractions. In Figure 6, *B* and *E*, the fraction of terminator readthrough was calculated by dividing the band intensity of readthrough transcripts by the total band intensity of terminated and readthrough transcripts.

### Reproducibility of the method

>10 independent preparations of ^C3-SC1^TECs were performed by S.L.K. and C.E.S. using separate reagent stocks and assessed by EMSA by using the final protocol (or a closely related protocol that yielded indistinguishable complexes). Examples of these data are shown in Figures 3 and S1*A*.

## Data availability

All data are contained in the manuscript as plotted values or representative gels. Source files in TIFF and/or gel format are available from the corresponding author (E.J.S.) upon request.

## Supporting information

This article contains supporting information.

## Conflict of interest

The authors have no conflicts of interest with the contents of this article.
