# Supplementary material for: Purification of synchronized Escherichia coli transcription elongation complexes by reversible immobilization on magnetic beads
Source: J Biol Chem. 2022 Mar 3;298(4):101789. doi: 10.1016/j.jbc.2022.101789 (PMC8969151; doi:10.1016/j.jbc.2022.101789)
Supplement: Supplemental Figures S1 and S2, Tables S1–S3 [file mmc1.pdf]

Purification of synchronized *E. coli* transcription elongation complexes by reversible immobilization on magnetic beads

Skyler L. Kelly, Courtney E. Szyjka, Eric J. Strobel

Materials Included:

Figure S1. Alternative conditions for $^{C3\text{-}SC1}$TEC purification.

Figure S2. Additional visualization of gels for $^{C3\text{-}SC1}$TEC kinetics and solid-phase transcription assays.

Table S1. Oligonucleotides used in this study.

Table S2. DNA template sequences.

Table S3. DNA templates prepared for this study.

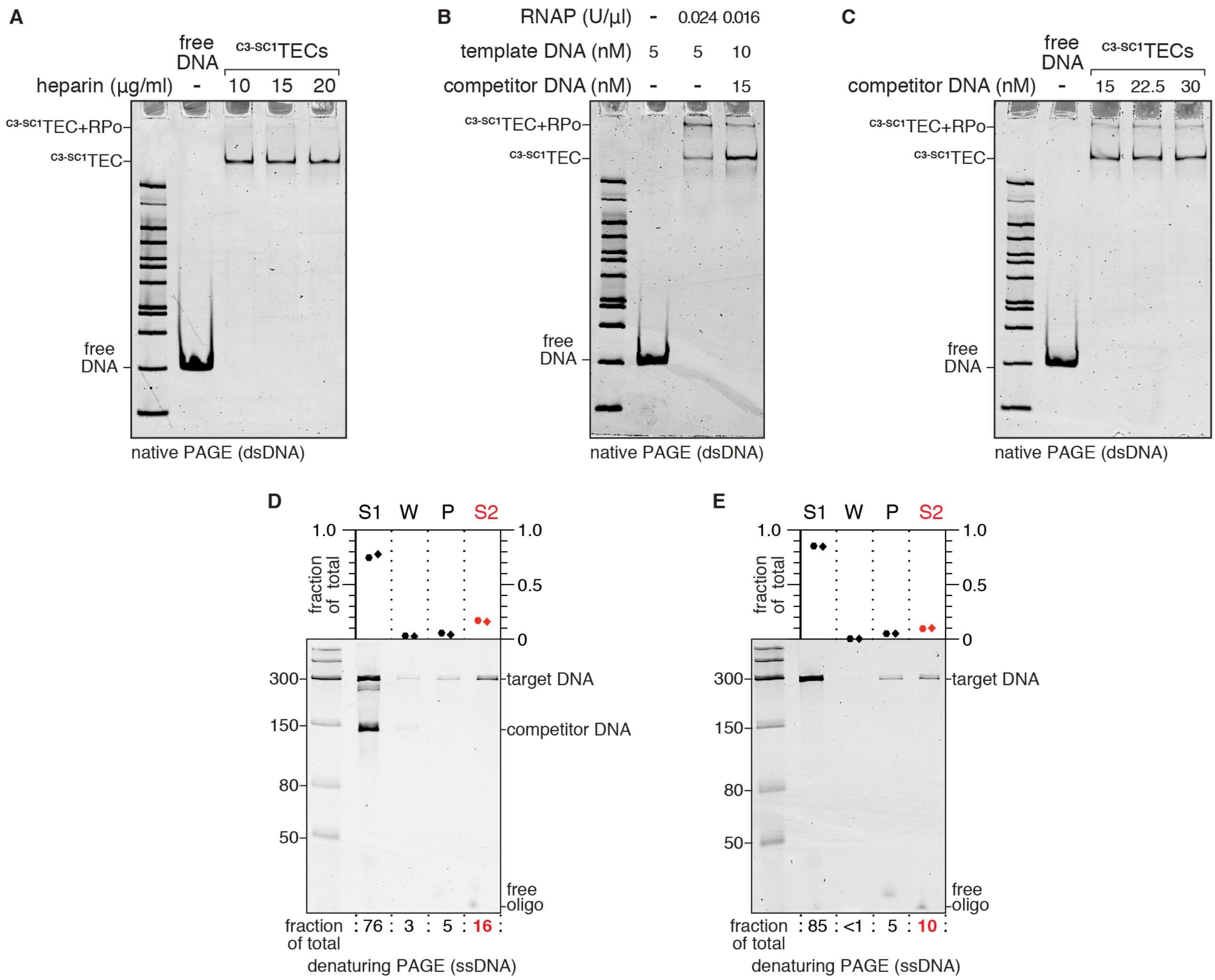

Figure S1. Alternative conditions for $^{C3\text{-}SC1}$TEC purification. *A*, EMSA of $^{C3\text{-}SC1}$TECs that were purified using variable amounts of heparin. *B*, EMSA of $^{C3\text{-}SC1}$TECs that were purified using a competitor DNA template instead of heparin. Omitting the competitor DNA and including excess RNAP favored the formation of slow-migrating complexes that contain both a $^{C3\text{-}SC1}$TEC and an open promoter complex. *C*, EMSA of $^{C3\text{-}SC1}$TECs that were purified using variable amounts of competitor DNA. *D*, denaturing PAGE analysis of purification fractions for $^{C3\text{-}SC1}$TECs that were prepared using 15 nM competitor DNA. *E*, denaturing PAGE analysis of purification fractions for $^{C3\text{-}SC1}$TECs that were prepared using 20 µg/ml heparin but without rifampicin.

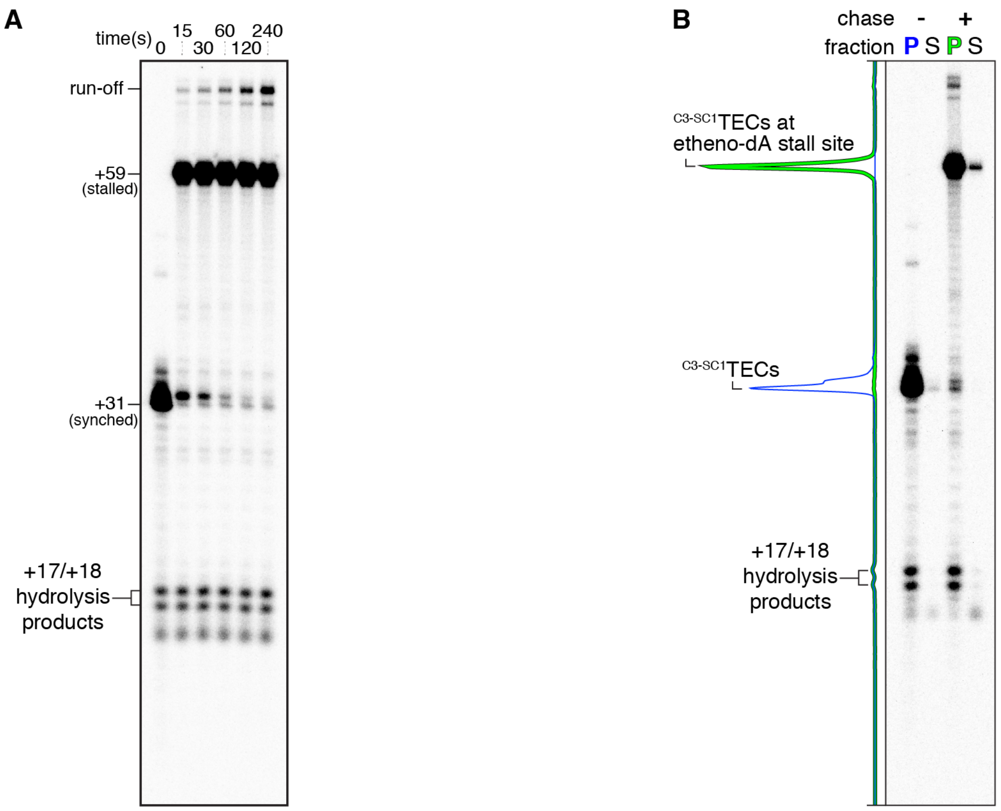

Figure S2. Additional visualization of gels for $^{C3\text{-}SC1}$TEC kinetics and solid-phase transcription assays. Gels from Figure 4A (*A*) and Figure 5A (*B*) are presented here with a darker grayscale setting to visualize trace RNAs.

Table S1. Oligonucleotides used in this study. Below is a table of oligonucleotides used in this study. The modification codes defined below are used for compatibility with Integrated DNA Technologies ordering. DNA containing internal etheno-dA requires an off-catalog order.

```
/iEth-dA/: internal etheno-dA
/3BioTEG/: 3' biotin-triethylene glycol
  /iSpPC/: internal photocleavable spacer
```

ID	Name	Sequence	Purif.
EJS017	dRP1_NoMod.R	AATGATACGGCGACCACCGAGATCTACACGTTCAGAGTTCTACAGTCCGACGATC	HPLC
TECD002	dRP1iEthdA.R	AATGATACGGCGACCACCGAGATCTACAC/iEth-dA/GTTCAGAGTTCTACAGTCCGACGATC	HPLC
TECD006	PRA1_NoMod.F	TTATCAAAAAGAGTATTGACTCTTTTACCTCTGGCGGTGATAATGGTTGCAT	HPLC
TECD018	Cap3_BioTEG	GAAGAGAAGAGCACCGAAT/3BioTEG/	HPLC
TECD019	Cap3_PCBioTEG	GAAGAGAAGAGCACCGAAT/iSpPC//3BioTEG/	HPLC
CES015	Cap3_NoMod	GAAGAGAAGAGCACCGAAT	None
TECD016	wlk29.tmp	ACCTCTGGCGGTGATAATGGTTGCATGGAGAGGGATATAGGGAAAGTGGTGGACGATCGTCGGACTGTAGAACTCTGAAC	PAGE

Table S2. DNA template sequences. Below is a table of containing the sequence of each DNA template. Fully annotated versions are available at Benchling (See 'DNA Template Sequences' in Experimental Procedures for hyperlinks).

Name	ID	Sequence
PRA1_Cap3_SC1_CbePfl_dRP1	1	ttatcaaaaagagtattgactcttttacctctggcggtgataatggttgcattcggtgctcttctcttcggccttcgggccaaattagatattagtcatatgactgacggaagtggagttaccacatgaagtatgactaggcatattatcttatatgccacaaaaagccgaccgtctgggcaaaaaaagcctggattgcgtcggcttttttatatggaaaagatcgtcggactgtagaactctgaacgtgtagatctcggtggtcgccgtatcatt
PRA1_Cap3_SC1_dRP1	2	ttatcaaaaagagtattgactcttttacctctggcggtgataatggttgcattcggtgctcttctcttcggccttcgggccaagatcgtcggactgtagaactctgaacgtgtagatctcggtggtcgccgtatcatt
PRA1_CbePfl_dRP1	3	ttatcaaaaagagtattgactcttttacctctggcggtgataatggttgcatattagatattagtcatatgactgacggaagtggagttaccacatgaagtatgactaggcatattatcttatatgccacaaaaagccgaccgtctgggcaaaaaaagcctggattgcgtcggcttttttatatggaaaagatcgtcggactgtagaactctgaacgtgtagatctcggtggtcgccgtatcatt
PRA1_Cap3_SC1_Cba2-2_dRP1	4	ttatcaaaaagagtattgactcttttacctctggcggtgataatggttgcattcggtgctcttctcttcggccttcgggccaaaataagagagtgtatctagggttccggtcaatagatgtctggtccgagcgatacaggatttcaatctacacttttaggaaaaaagcctaaaggacgagtctctgcaaagagatttgttcttgggctttatttttttatctttatttaagatcgtcggactgtagaactctgaacgtgtagatctcggtggtcgccgtatcatt
PRA1_Cba2-2_dRP1	5	ttatcaaaaagagtattgactcttttacctctggcggtgataatggttgcataataagagagtgtatctagggttccggtcaatagatgtctggtccgagcgatacaggatttcaatctacacttttaggaaaaaagcctaaaggacgagtctctgcaaagagatttgttcttgggctttatttttttatctttatttaagatcgtcggactgtagaactctgaacgtgtagatctcggtggtcgccgtatcatt
wlk29_Competitor	6	ttatcaaaaagagtattgactcttttacctctggcggtgataatggttgcatggagagggatatagggaaagtggtggacgatcgtcggactgtagaactctgaacgtgtagatctcggtggtcgccgtatcatt

Table S3. DNA templates prepared for this study. Below is a table of DNA templates that were prepared for this study, including the primers, plasmids, and template oligos used, DNA modifications, the PCR polymerase used, whether translesion synthesis was performed, which reaction clean up protocol was used (see Experimental Procedures), and the figures in which each DNA template was used.

ID	Fwd Primer	Rev Primer	Template	Modifications	PCR Polymerase	Translesion Synthesis	Clean Up	Used in Fig(s)
1	TECD006	EJS017	pCES002	none	Vent (exo-)	N/A	Gel extracted	2, 3B, 3C, 6B, 6C, S1A, S1B, S1C, S1D, S1E
2	TECD006	TECD002	Gel-purified linear DNA from pCES003	Int etheno-dA	Q5	Yes	PCR clean-up	4, 5, S2A, S2B
3	TECD006	EJS017	pCES004	none	Vent (exo-)	N/A	Gel extraction	6B, 6C
4	TECD006	EJS017	pCES005	none	Vent (exo-)	N/A	Gel extraction	6E, 6F
5	TECD006	EJS017	pCES006	none	Vent (exo-)	N/A	Gel extraction	6E, 6F
6	TECD006	TECD002	TECD016	Int etheno-dA	Q5	Yes	PCR clean-up	S1B, S1C, S1D
